# Posterior subhyaloid precipitates in cytomegalovirus retinitis

**DOI:** 10.1007/s12348-011-0032-z

**Published:** 2011-07-29

**Authors:** Raquel Goldhardt, Ninel Z. Gregori, Thomas Albini, Shalini Yalamanchi, Andres Emanuelli

**Affiliations:** 1 Ophthalmology Section, Miami Veterans Affairs Medical Center, 1201 NW 16th Ave, Miami, FL 33125 USA; 2 Department of Ophthalmology, Bascom Palmer Eye Institute, University of Miami Miller School of Medicine, Miami, FL USA

**Keywords:** Cytomegalovirus retinitis, CMV, Posterior subhyaloid precipitates, Inflammatory precipitates, Uveitis, AIDS, Optical coherence tomography, OCT

## Abstract

**Purpose:**

This study aims to report a novel finding of posterior subhyaloid precipitates (PSPs) in two patients with cytomegalovirus (CMV) retinitis.

**Methods:**

A small case series was conducted.

**Results:**

Clinical findings, treatment, and follow-up of two patients with CMV and PSPs are presented.

**Conclusions:**

Inflammatory precipitates may collect in the posterior subhyaloid space in acute CMV retinitis and resolve with treatment.

The AIDS epidemic has provided an opportunity to study the course and clinical features of cytomegalovirus (CMV) retinitis, which is a leading cause of blindness in these immunocompromised patients. The CMV retinitis lesions may develop in all areas of the retina, although the majority of early lesions are adjacent to blood vessels [1]. Secondary rhegmatogenous retinal detachment (RD) often leads to severe vision loss and occurs in 15–40% of eyes at some point during the course of the disease [2, 3]. Mild vitreous and anterior chamber inflammatory reactions are almost invariably present in a patient with active CMV retinitis, but moderate to severe intraocular inflammation is uncommon [4]. We describe two patients in whom a novel finding of posterior subhyaloid precipitates (PSPs) was documented with fundus photography and spectral-domain optical coherence tomography (OCT).

## Case presentation 1

**Fig. 1:**
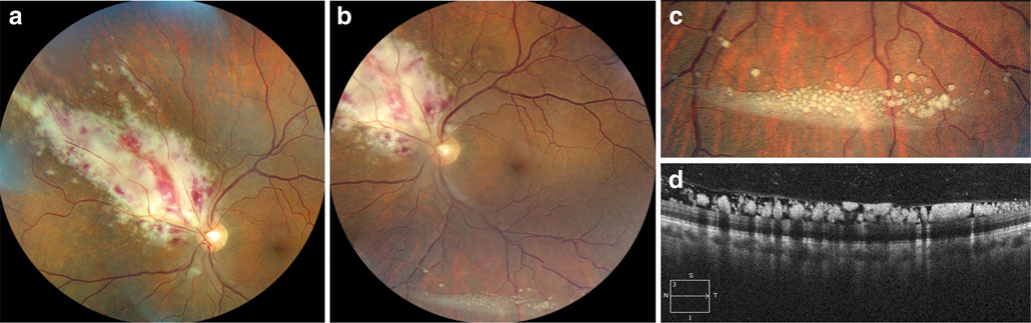
**a** Superonasal CMV retinitis lesion with retinal granular necrotic appearance and few hemorrhages at the time of diagnosis. **b** Two weeks after diagnosis, inferior posterior subhyaloid precipitates (PSPs) were observed. **c** Fundus photo showing the PSPs below inferotemporal arcade. **d** Spectral-domain optical coherence tomography showing multiple PSPs

A 50-year-old HIV-positive African-American male, with CD4 count of 46 cells/ml, presented with sudden floaters in the left eye. Visual acuity was 20/20 in the right eye and 20/25 in the left eye. Slit lamp examination demonstrated clear cornea in both eyes, no anterior chamber cells in the right eye, and rare cells in the left eye. The lens was clear in both eyes. Fundus examination of the left eye revealed a large 7 × 4 disk diameter (DD) irregular lesion superonasal to the optic nerve consisting of retinal whitening with granular appearance and characteristic isolated “satellite” lesions along its edge. Few hemorrhages were present within the lesion. Minimal vitreous inflammation was present over the involved retina (Fig. 1a). The diagnosis of cytomegalovirus retinitis was made based on the fundus examination and clinical history. The patient was admitted for induction therapy with intravenous ganciclovir, 5 mg/kg, and was re-started on highly active antiretroviral therapy (HAART) therapy.

**Fig. 2:**
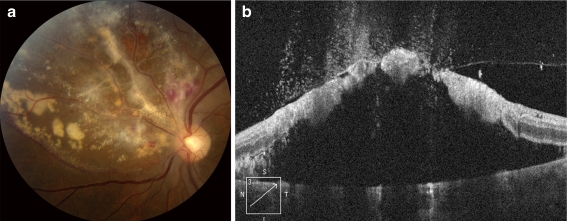
**a** Fundus photo showing thinned retina and RD. **b** Showing the posterior hyaloid attached to the retina with overlying inflammation

One week later, white granular subhyaloid opacities located 2 DD below the inferotemporal arcade and away from the main CMV lesion were observed on fundus examination (Fig. 1b, c). A spectral-domain optical coherence tomography (SD-OCT) demonstrated hyperreflective round opacities clearly located in the space between the posterior hyaloid and the retina. (Fig. 1d). After 10 days of induction with intravenous ganciclovir, the patient was switched to oral valganciclovir 900 mg twice per day. Twelve days after presentation, the superonasal CMV lesion matured into a grayish gliotic scar with several atrophic retinal holes and a localized RD. One month after the diagnosis of CMV and 2 weeks after the appearance of the RD, the SD-OCT showed that the posterior hyaloid was still attached to the retina over the area of CMV lesion, bridging the retinal holes and keeping a localized rhegmatogenous RD stable (Fig. 2a, b).

**Fig. 3:**
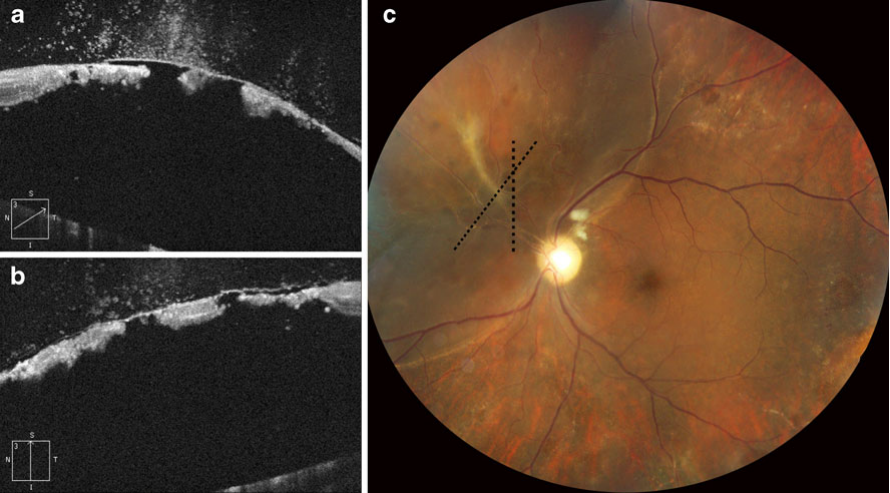
**a**, **b** Four months after the diagnosis, spectral-domain OCT cuts through the superonasal CMV lesion demonstrate detached thin necrotic retina with multiple discontinuities and persistent hyaloidal attachment bridging the retinal holes. **c** Fundus photography demonstrates two small new necrotic retinal lesions above the optic nerve and resolution of the inferior PSPs

**Fig. 4:**
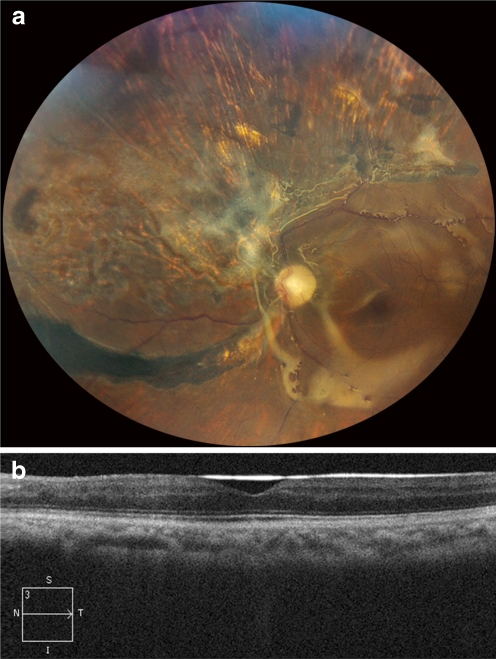
**a** Fundus photos 2 months after PPV with silicone oil and ganciclovir implant. **b** OCT showing attached macula

Four months after the diagnosis, while still on oral valganciclovir maintenance dose, the localized RD remained stable with attached posterior hyaloid (Fig. 3a, b). The PSPs resolved, but a new small necrotic lesion appeared superior to the optic nerve. A week later, another satellite lesion developed, and the patient’s oral valganciclovir dose was increased from 900 mg daily to 900 mg twice daily. In addition, an intravitreal ganciclovir injection (2 mg/0.05 ml) was administered (Fig. 3c). CD4 was 57/mm^3^. The vision in the left eye was stable at 20/25. One week after intravitreal injection of ganciclovir, the RD progressed threatening the macula and lens-sparing pars plana vitrectomy (PPV) with silicone oil and ganciclovir implant (Vitrasert) was performed. Four months after the surgery, vision was 20/25 and retina was attached under silicone oil (Fig. 4a, b). CD4 increased to 125/mm^3^.

## Case presentation 2

A 41-year-old healthy white female presented with blurry vision and floaters in the right eye for 4 weeks. She was referred by an outside ophthalmologist for possible acute retinal necrosis and was initiated on valacyclovir hydrochloride (Valtrex) 1 g three times daily. The patient had a history of three episodes of shingles involving her scalp, right torso, and leg. The most recent episode occurred 2 months prior. She also had several episodes of recurrent pneumonia. She denied any prior ocular history.

**Fig. 5:**
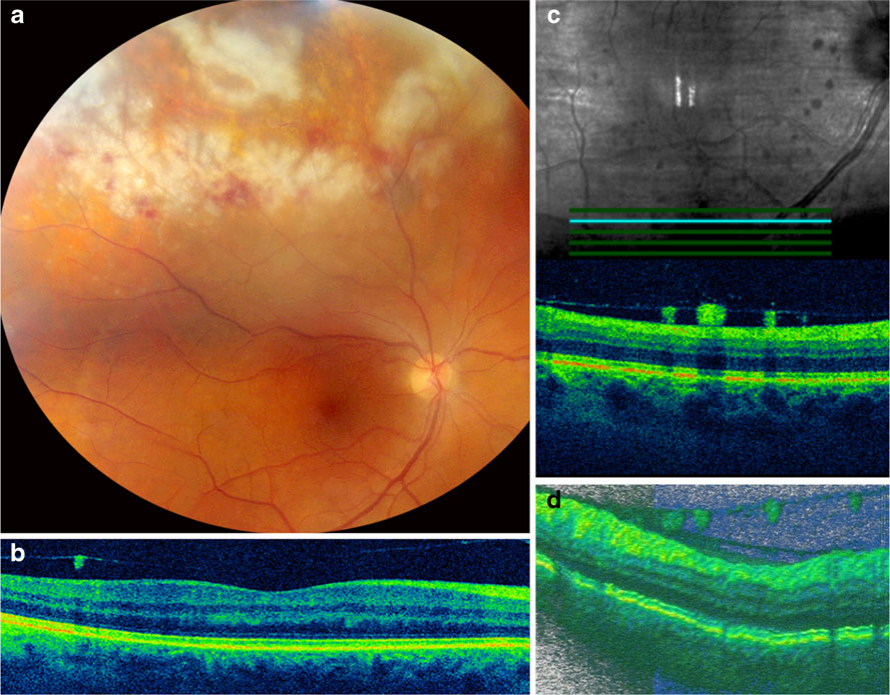
**a** Right eye with extensive retinal necrotic lesion with overlying hemorrhages. **b** SD-OCT image showing a normal macula. **c**, **d** SD-OCT demonstrating the posterior subhyaloid precipitates

Best corrected visual acuity was 20/25 OD and 20/20 OS. Slit lamp examination demonstrated diffuse white keratic precipitates in the right eye with 2+ anterior chamber cell and no cell in the left eye. The lens was clear in both eyes. Dilated fundus examination showed 3+ vitreous cell in the right eye and extensive retinal necrosis with whitening and hemorrhage superiorly (Fig. 5a). Fundus evaluation of the left eye was unremarkable.

OCT evaluation of the right eye demonstrated no evidence of fluid in the macula, but demonstrated the presence of PSPs overlying the retina away from the retinitis lesion, along the inferotemporal arcade (Fig. 5b–d).

**Fig. 6:**
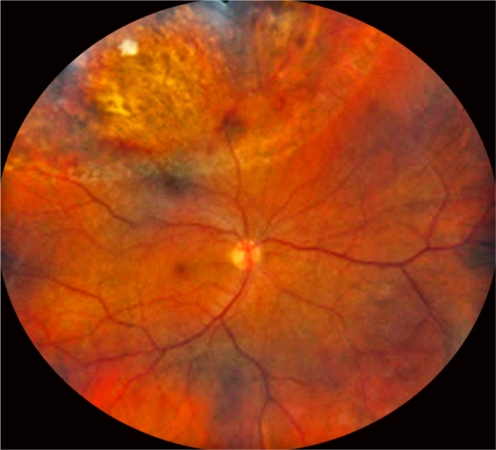
Laser demarcation is observed with residual whitening and retinal hemorrhages

The patient underwent an anterior chamber tap for viral PCR studies and was treated with intravitreal ganciclovir 2 mg/0.05 ml. PCR revealed CMV. HIV test was negative. Three weeks after presentation, she had received a total of four intravitreal ganciclovir injections and barricade laser superiorly by her local ophthalmologist. Best corrected visual acuity was 20/25 OD and 20/20 OS. The keratic precipitates decreased and trace anterior chamber cell was present in the right eye. Fundus examination showed laser demarcating and improved retinal necrosis with pigmentary changes and mild residual whitening and hemorrhages (Fig. 6). The patient was continued on oral valganciclovir 900 mg twice daily and was scheduled for an immunology evaluation.

## Discussion

We present two cases showing a peculiar finding of PSPs occurring in CMV retinitis. Such precipitates have not been previously well documented and reported; therefore, the fundus photos and spectral-domain OCTs presented here are unique in the literature. The OCTs clearly show that these precipitates are located between the posterior hyaloid and the retina and therefore appear to be distinct from vitreous precipitates, which have been previously reported. The curvilinear organization of PSPs in the posterior segment suggest that they descent with gravity and collect along the attachment of the posterior hyaloid to underlying retina. These PSPs cleared during the course of treatment and therefore may potentially be followed as another clinical sign of response to therapy. We can speculate that because the PSPs clear with treatment, they are most likely made up of inflammatory cells. In conclusion, we present a novel finding of posterior subhyaloid precipitates, which were clearly documented by the spectral-domain OCT. We expect that these cases will contribute to the further characterization of the CMV retinitis manifestation in the era of HAART and OCT imaging.
